# Survival in patients with Parkinson’s disease: a ten-year follow-up study in northern China

**DOI:** 10.1186/s12883-022-02899-5

**Published:** 2022-09-22

**Authors:** Song Wang, Tao Li, Tingting Zhou, Lanlan Pu, Hai-Yang Wang, Xiaoxue Yin, Xinqing Hao, Lu Ren, Zhanhua Liang

**Affiliations:** 1 Department of Neurology, First Affiliated Hospital of Dalian Medical University, No.222, Zhongshan Road, Dalian, 116011 Liaoning Province China; 2 Department of Neurology, Jining No. 1 People’s Hospital, Jining, 272000 China

**Keywords:** Parkinson’s disease, Survival, Prognostic factors, Mortality, Standardized mortality ratio

## Abstract

**Background:**

A thorough understanding of the factors that influence patient survival in Parkinson’s disease (PD) will aid in prognosis prediction and provide a new direction for disease modification treatment. Currently, there are no standardized mortality ratio (SMR) data for PD patients in the northern Chinese mainland. The main focus of this study was to determine which factors in the prospectively collected baseline characteristics can affect the survival of PD patients. In addition, for the first time, we investigated the SMR of PD patients in northern China.

**Methods:**

Between 2009 and 2012, 218 PD patients were continuously recruited from the movement disorder clinic of the First Affiliated Hospital of Dalian Medical University and followed up until death or May 31, 2021. The prespecified prognostic variables were demographics, clinical features, lifestyle factors, and drug dose prospectively collected at baseline. To determine the independent predictors of survival during follow-up, the Cox proportional hazards model was used. Kaplan–Meier analysis was applied to estimate the overall survival curve and to compare survival between layers based on statistically significant predictors. The SMR of this northern Chinese mainland PD cohort was calculated.

**Results:**

After a mean follow-up of 9.58 ± 2.27 years, 50 patients (22.90%) died. Factors that could individually predict shortened survival during follow-up included older age at onset (hazard ratio [HR] 1.10, 95% confidence interval [CI] 1.06–1.15), Hoehn and Yahr (H&Y) stage ≥ 3 (HR 9.36, 95% CI 2.82–31.03) and severe cognitive impairment (HR 6.18, 95% CI 2.75–13.88). Univariate Cox regression revealed that a certain amount of physical activity was associated with better survival (HR 0.41, 95% CI 0.22–0.74), while fatigue was associated with an increased risk of death (HR 2.54, 95% CI 1.37–4.70). The overall SMR was 1.32 (95% CI 0.98–1.74).

**Conclusions:**

Older age at onset, higher baseline H&Y stage, and severe cognitive impairment have a negative impact on survival. The 10-year survival of PD patients is not significantly different from that of the general population in China.

**Supplementary Information:**

The online version contains supplementary material available at 10.1186/s12883-022-02899-5.

## Background

Parkinson’s disease (PD) is a neurodegenerative disease that occurs mainly in the middle-aged and elderly population. With aging of the population and an increase in life expectancy, the health burden of this disease is rising sharply worldwide. It is estimated that, by 2030, global PD patients will increase from 4.1 million in 2005 to 8.7 million, of which the number in China will reach 4.94 million [1]. Several studies have suggested that the survival of PD patients is poorer than that of the general population [2]. Investigating the survival of PD patients is helpful to understand the disease burden to provide the basis for patient care, family support and medical security budgets. Identifying the factors that affect the survival of PD patients can help to better predict the prognosis in clinical practice, and among them, the intervenable factors may provide a novel direction for disease modification treatment of PD.

Current studies on factors affecting the survival of PD patients involve four aspects: demographics, clinical characteristics, intervention measures and environmental factors [2]. Among them, the recognized risk factors include older age at onset and cognitive impairment [3–5]. However, many shortcomings or controversial areas remain to be further explored. First, studies of prognostic factors, such as sex, severity of motor symptoms, hallucinations, depression, autonomic dysfunction, smoking, and deep brain stimulation (DBS) treatment, have shown inconsistent results [2, 5–14]. Furthermore, it remains unclear whether the presence of fatigue is an independent predictor of the decline in the survival of PD patients. Finally, lifestyle factors, such as caffeine intake, moderate drinking, and exercise intervention, may prolong the survival of PD patients [15, 16], this has yet to be confirmed by repeated data.

The standardized mortality ratio (SMR) is a commonly used indicator in survival analyses. In Spain, Italy and the United States, the SMR of PD patients is higher in northern regions than in southern regions [17–19]. However, a study in Japan showed that the mortality is higher in the southwest than in the northeast [20]. The Chinese mainland is divided into the south and north by the Qinling-Huaihe line. Currently, only Shanghai (SMR = 0.87, 95% CI 0.59–1.25) [6] and Hong Kong (SMR = 1.10, 95% CI 0.80–1.50) [4] in the southern region have conducted survival surveys for more than ten years. No SMR data are available for PD patients in the northern region of the Chinese mainland.

The primary objective of our study was to determine which factors included in the prospectively collected baseline information (demographics, clinical characteristics, lifestyle factors, and drug regimen) affect survival in PD patients. Furthermore, the SMR of a given cohort of PD patients in the northern Chinese mainland was calculated to explore whether PD affects survival. Finally, the causes of death in this PD cohort were investigated.

## Materials and methods

### Study participants

From October 2009 to October 2012, we continuously recruited all patients with new and existing PD diagnoses who permanently settled in Dalian and received follow-up visits in the movement disorder clinic of the First Affiliated Hospital of Dalian Medical University. The movement disorder clinic is the only tertiary referral center for PD in Dalian, with referrals from neurologists, family physicians, community doctors, general practitioners and emergency department doctors. The diagnosis of idiopathic Parkinson’s disease was carried out by experienced movement disorder specialists based on the United Kingdom Parkinson’s Disease Society Brain Bank criteria [21]. Participants were excluded under the following conditions: (1) hallucinations or dementia occurred within one year after the onset of disease; (2) development of features that may lead to a diagnosis other than idiopathic PD; and (3) incomplete clinical information.

The study was approved by the Ethics Committee of the First Affiliated Hospital of Dalian Medical University, China. Written informed consent was obtained from each participant.

### Clinical assessments

Information collection and clinical evaluation at baseline were performed by a single trained movement disorder specialist. The standardized questionnaires recorded the following information: (1) sociodemographic characteristics (age, gender, education, occupation); (2) medical history, family history and treatment history of PD; (3) clinical comorbidities (such as hypertension, diabetes, heart disease, and stroke); and (4) lifestyle factors (smoking history; average intake of caffeinated tea, caffeinated coffee and alcohol in adulthood; and physical exercise level in the past ten years). Clinical assessments were performed by using measurement instruments considered to be effective and reliable in PD, including the Hoehn and Yahr (H&Y) stage, Unified Parkinson Disease Rating Scale (UPDRS), 30-item Nonmotor Symptoms Questionnaire (NMSQ-30), Hamilton Depression Scale (HAMD), Mini-mental State Examination (MMSE), Pittsburgh Sleep Quality Index (PSQI), and Fatigue Severity Scale (FSS). All the above evaluations were conducted in On medication, and all data were validated by senior specialists.

### Potential predictors for survival

Potential predictors were prespecified, including age at onset, sex, education, presence of clinical comorbidities, and whether tremor was an initial symptom. The severity of PD was based on the H&Y stage, and the symmetry of motor signs was defined as the absence of left–right differences in neurological examinations or bilateral onset. PD was classified into three motor-based subtypes: postural instability/gait difficulty (PIGD), tremor dominant (TD), and indeterminate, which were based on the TD/PIGD ratio calculated from the corresponding items on the UPDRS scale (PIGD ≤ 1.0, TD ≥ 1.5, indeterminate = 1.0–1.5) [22]. Dysphagia was considered to be present if the UPDRS II question 3 scored1 or higher. Cognitive status was based on the MMSE score and given different cutoff values according to the level of education, which was divided into three groups: normal, mildly impaired and severely impaired. Patients were classified as having hallucinations if UPDRS I question 2 scored 2 or higher. The subjects were assessed for depression using the HAMD: ≤ 8 for no depression, > 8–20 for mild depression, > 20–35 for moderate depression, and > 35 for severe depression. Constipation was considered to be present if the answer to question 5 of the NMSQ-30 was “yes” [23]. Patients were classified as having fatigue if the FSS score was above 4 points [24]. According to the PSQI score, sleep quality was divided into four grades (very good, fairly good, fairly bad, very bad). The levodopa equivalent daily dosage (LEDD) was calculated according to the method proposed in a previous study [25].The following criteria were adopted for the classification of lifestyle factors: (1) participants were divided into groups of never, former and current smokers based on self-reported smoking history. Meanwhile, we adopted pack-years for quantitative analysis, where one unit corresponded to one pack of cigarettes per day during the year; (2) participants were required to report whether they had consumed caffeinated tea, caffeinated coffee, beer, or liquor and, if so, to record when they had started and stopped drinking. The average frequency (daily/weekly/monthly) and the average amount consumed per time (one cup = 150 ml) were recorded. Subjects with a history of coffee and/or tea drinking were also asked to report beverage strength (strong/medium/weak, assigned 2, 1, 0.5, respectively). Alcohol intake was counted based on liquor (one unit of liquor = ten units of beer). We derived the weighted average number of beverages consumed per day from age 18 to the enrollment date for each beverage and calculated the median beverage-specific intake among consumers. Participants were further categorized into three groups: never drinkers, drinking less than and equal to/more than the median intake per day; (3) participants were also asked to report whether they had exercised intentionally during their leisure time in the past decade and, if so, to record the average number of hours per day and days per week they had exercised mildly, moderately or vigorously. These data were used to obtain the metabolic equivalent hours per week (MET-h/wk) of physical exercise, and the median among exercisers was also calculated. Participants were then divided into three groups: never exercisers, exercising less than and at/above the median.

### Ascertainment of vital status

Vital status follow-up began at the date of the baseline examination and continued until death or 31 May 2021. The date and cause of death were determined from multiple sources, including medical records, autopsy reports and death certificates, and were cross-checked through visits to participants’ family and friends. The leading causes of death were classified into eight categories based on the tenth revision of the International Classification of Diseases (ICD-10). If the patient underwent DBS surgery during the ten-year follow-up period, the timing of the surgery was verified by reviewing hospital records and documented.

### Statistical analyses

Descriptive statistics were applied to describe baseline demographics, lifestyle factors, and clinical characteristics. Continuous variables were described as the means (± standard deviation [SD]) and medians (range), while categorical variables were expressed as the absolute numbers and percentages.

The primary objective was to investigate potential predictors of survival in PD patients. Survival time was calculated from the date of enrollment for each subject. Cox proportional hazards (PH) analysis was used to investigate the effect of prespecified variables on survival time. In addition to the age at onset, LEDD and pack-years of smoking as continuous variables, the remaining variables were input to the model as categorical variables. Baseline variables deemed clinically relevant or with a univariate relationship to outcome were entered into multivariate Cox regression model. Given the number of events available, variables for inclusion were carefully chosen to ensure concision of the final model. To avoid multicollinearity, we examined the correlation between variables and excluded possible intermediary variables from the model. Kaplan–Meier analysis was used to describe the overall survival curve and to compare survival between layers based on statistically significant predictors. The PH assumption was tested by adding time-dependent covariates to the model and visually inspecting the Kaplan–Meier survival curve and the log minus log function.

The secondary objective was to calculate the SMR and the corresponding 95% confidence intervals (CIs). SMR was calculated as the ratio of observed to expected deaths. According to the China Demographic and Employment Statistics Yearbook (2011–2020), the age- and sex-specific mortality of the Chinese population during 2011–2020 was applied to the cohort of this study to obtain the expected number of deaths. The 95% CI for SMR was estimated by Byar’s method assuming that different death categories followed a Poisson distribution. SMR and corresponding 95% CIs were calculated separately for the overall cohort and for patients who opted for DBS treatment during the follow-up.

All statistical analyses were performed using IBM SPSS Statistics for Windows version 25.0 (IBM Corporation, Armonk, NY, USA). Two-tailed *P* values < 0.05 were considered statistically significant.

## Results

**Fig. 1:**
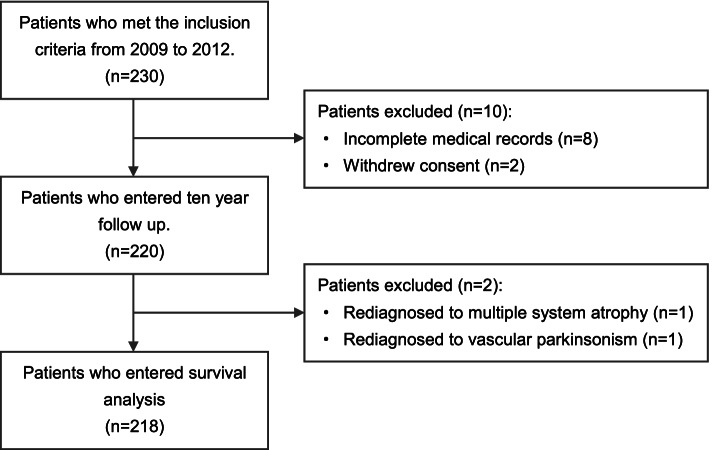
Details of the patient cohort follow-up from 2009 to 2021

We initially recruited 230 patients, eight of whom had incomplete clinical information, and two who withdrew consent. Two subjects (one with a revised diagnosis of multiple system atrophy and one with vascular Parkinson’s syndrome) were further diagnosed during follow-up and excluded. Overall, 218 patients with idiopathic PD were ultimately identified for survival analysis. Figure 1 details the patients’ follow-up.

Baseline characteristics and vital status at the end of follow-up are presented in Table 1. Among the 218 PD patients included in the final analysis, the mean age of disease onset was 56.89 ± 9.12 years (range 30.00–79.00 years), and the mean course of disease at enrollment was 4.01 ± 3.61 years (range 0.32–24.31 years). One hundred and sixteen (53.20%) patients were women. As of 31 May 2021, the mean duration of follow-up was 9.58 ± 2.27 years (median 10.31, range 0.47–11.65 years), with follow-up of the deceased continuing until the time of death. When the mean FSS score > 4 was considered as the critical point, the incidence of fatigue was 52.80%. In the analysis of lifestyle factors, 150 patients (68.80%) participated in intentional physical exercise during leisure time. Twenty-one (9.60%), 52 (23.90%) and 37 (17.00%) patients reported histories of caffeinated coffee, caffeinated tea, and alcohol consumption, respectively. Forty-two patients (19.30%) reported a history or current history of smoking, with an average of 4.76 ± 13.40 pack-years (range 0.00–96.00 pack-years). In addition, 90 patients (41.30%) in our cohort opted for DBS surgery during the ten-year follow-up.

**Table 1:** Baseline characteristics and vital status at the end of follow-up

Baseline variable	No. (%); mean [± SD]; Median {range}	End Point Vital Status, Deceased/Alive, No
Age at onset, y	56.89 [± 9.12]; 57.00 {30.00–79.00}	63.10 [± 8.20]/55.05 [± 8.57]
Disease duration, y	4.01 [± 3.61]; 2.99 {0.32–24.31}	3.85 [± 3.66]/4.06 [± 3.60]
LEDD, mg	358.60 [± 204.24]; 300.00 {0.00–950.00}	387.50 [± 218.02]/350.00 [± 199.83]
Smoking history, pack-years	4.76 [± 13.40]; 0.00 {0.00–96.00}	6.75 [± 15.14]/4.17 [± 12.83]
Sex		
Female	116 (53.20)	26/90
Male	102 (46.80)	24/78
Clinical comorbidity		
No	128 (58.70)	23/105
Yes	90 (41.30)	27/63
Tremor as initial symptom		
No	89 (40.80)	20/69
Yes	129 (59.20)	30/99
Symmetry of motor signs		
No	183 (83.90)	33/150
Yes	35 (16.10)	17/18
H&Y stage		
< 3	213 (97.70)	46/167
≥ 3	5 (2.30)	4/1
Motor-based subtype		
TD	131 (60.10)	20/111
Indeterminate	19 (8.70)	5/14
PIGD	68 (31.20)	25/43
Dysphagia		
No	177 (81.20)	30/147
Yes	41 (18.80)	20/21
Education		
Illiteracy	5 (2.30)	1/4
Primary school	40 (18.30)	10/30
Middle or high school	148 (67.90)	33/115
University or higher	25 (11.50)	6/19
Cognitive impairment^a^		
None	147 (67.40)	22/125
Mild	54 (24.80)	13/41
Severe	17 (7.80)	15/2
Hallucinations		
No	206 (94.50)	46/160
Yes	12 (5.50)	4/8
Depression		
None	90 (41.30)	17/73
Mild	105 (48.20)	24/81
Moderate	23 (10.60)	9/14
Severe	0 (.00)	0/0
Constipation		
No	92 (42.20)	16/76
Yes	126 (57.80)	34/92
Fatigue		
No	103 (47.20)	14/89
Yes	115 (52.80)	36/79
Sleep quality grade		
Very good	108 (49.50)	16/92
Fairly good	70 (32.10)	20/50
Fairly bad	34 (15.60)	12/22
Very bad	6 (2.80)	2/4
Smoking history		
Never	176 (80.70)	37/139
Former	27 (12.40)	10/17
Current, at baseline	15 (6.90)	3/12
Caffeinated coffee		
Never	197 (90.40)	48/149
< median (cups/day)^b^	10 (4.60)	1/9
≥ median (cups/day)^b^	11 (5.00)	1/10
Caffeinated tea		
Never	166 (76.10)	43/123
< median (cups/day)^b^	26 (11.90)	5/21
≥ median (cups/day)^b^	26 (11.90)	2/24
Alcohol		
Never	181 (83.00)	38/143
< median (cups/day)^b^	18 (8.30)	7/11
≥ median (cups/day)^b^	19 (8.70)	5/14
Physical exercise at leisure time		
Never	68 (31.20)	24/44
< median (MET-h/wk)	34 (15.60)	7/27
≥ median (MET-h/wk)	116 (53.20)	19/97

^a^Cognitive status was based on the MMSE score, and we used different demarcation criteria according to educational background: University or higher (normal, 28–30; mildly impaired, 24–27; and severely impaired ≤ 23); Middle or high school (normal, 27–30; mildly impaired, 23–26; and severely impaired ≤ 22); Primary school (normal, 25–30; mildly impaired, 21–24; and severely impaired ≤ 20); and Illiteracy (normal, 22–30; mildly impaired, 18–21; and severely impaired ≤ 17); ^b^One cup = 150 ml

*SD* standard deviation, *LEDD* levodopa equivalent daily dosage, *H&Y* Hoehn and Yahr stage, *TD* tremor-dominant, *PIGD* posture instability gait difficulty-dominant

Univariate Cox regression showed that factors associated with better survival included above-median physical exercise (HR 0.41, 95% CI 0.22–0.74) and DBS therapy (HR 0.44, 95% CI 0.24–0.83), while fatigue was associated with an increased risk of death (HR 2.54, 95% CI 1.37–4.70). Other factors associated with a higher risk of death included older age at onset, symmetrical motor signs at onset, H&Y stage ≥ 3, PIGD motor subtype, dysphagia, severe cognitive impairment and poor sleep quality (Table 2). Factors not significantly related to survival in univariate analysis are listed in Table S1.

**Table 2:** Associations of baseline variables with survival as determined by Cox proportional hazards models

Baseline variable	Univariate Cox regression^a^		Multivariate Cox regression	
	Hazard ratio (95% CI)	*P* Value	Hazard ratio (95% CI)	*P* Value
Age at onset,1-y increase	1.11 (1.07–1.15)	< 0.001	**1.10 (1.06–1.15)**	** < 0.001**
Symmetry of motor signs				
No	1.00 (reference)		1.00 (reference)	
Yes	3.24 (1.80–5.82)	< 0.001	1.34 (0.69–2.60)	0.384
H&Y stage				
< 3	1.00 (reference)		1.00 (reference)	
≥ 3	7.63 (2.71–21.43)	< 0.001	**9.36 (2.82–31.03)**	** < 0.001**
Motor-based subtype				
TD	1.00 (reference)		1.00 (reference)	
Indeterminate	1.81 (0.68–4.81)	0.238	1.27 (0.47–3.44)	0.639
PIGD	2.71 (1.50–4.88)	0.001	1.41 (0.70–2.84)	0.338
Dysphagia				
No	1.00 (reference)		1.00 (reference)	
Yes	3.47 (1.97–6.12)	< 0.001	1.47 (0.77–2.81)	0.240
Cognitive impairment				
None	1.00 (reference)		1.00 (reference)	
Mild	1.76 (0.89–3.50)	0.106	1.41 (0.66–3.04)	0.378
Severe	12.77 (6.51–25.04)	< 0.001	**6.18 (2.75–13.88)**	** < 0.001**
Fatigue				
No	1.00 (reference)		1.00 (reference)	
Yes	2.54 (1.37–4.70)	0.003	1.36 (0.68–2.73)	0.386
Sleep quality grade^b^				
Very good	1.00 (reference)			
Fairly good	2.09 (1.09–4.04)	0.028		
Fairly bad	2.87 (1.36–6.07)	0.006		
Very bad	2.52 (0.58–10.95)	0.219		
Physical exercise at leisure time				
Never	1.00 (reference)		1.00 (reference)	
< median	0.52 (0.22–1.20)	0.126	0.74 (0.31–1.79)	0.505
≥ median	0.41 (0.22–0.74)	0.003	0.70 (0.36–1.35)	0.285
Deep brain stimulation				
No	1.00 (reference)		1.00 (reference)	
Yes	0.44 (0.24–0.83)	0.012	0.57 (0.30–1.12)	0.103

^a^Only statistically significant variables are listed in the table, and other variables are showed in Table S1; ^b^Sleep quality grade is significantly correlated with multiple variables, given the possible intermediary variable it is, we excluded it from multivariate Cox regression model to avoid multicollinearity

*CI* Confidence interval, *H&Y* Hoehn and Yahr stage, *TD* Tremor-dominant, *PIGD* Posture instability gait difficulty-dominant

**Fig. 2:**
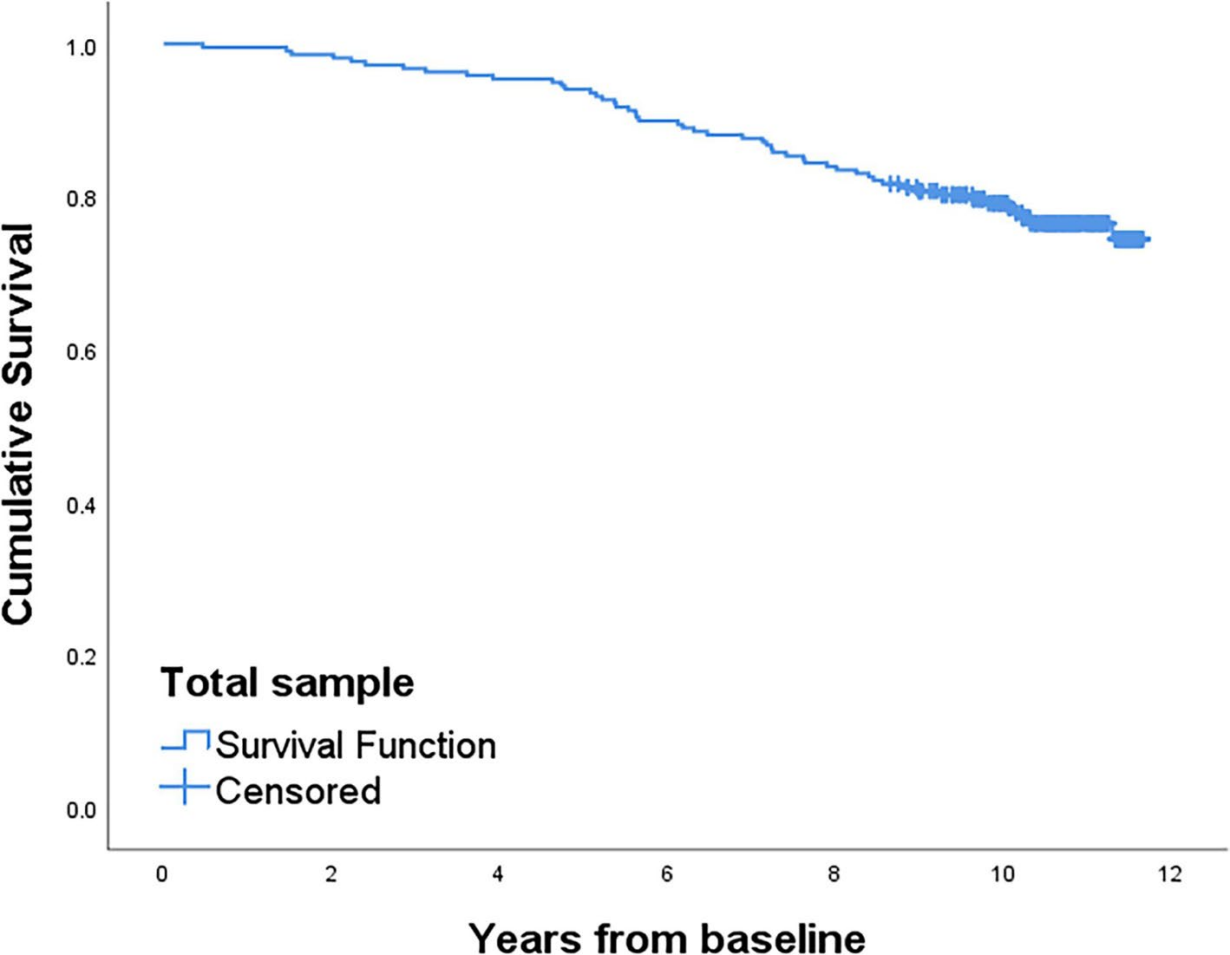
Kaplan–Meier plot of total sample

**Fig. 3:**
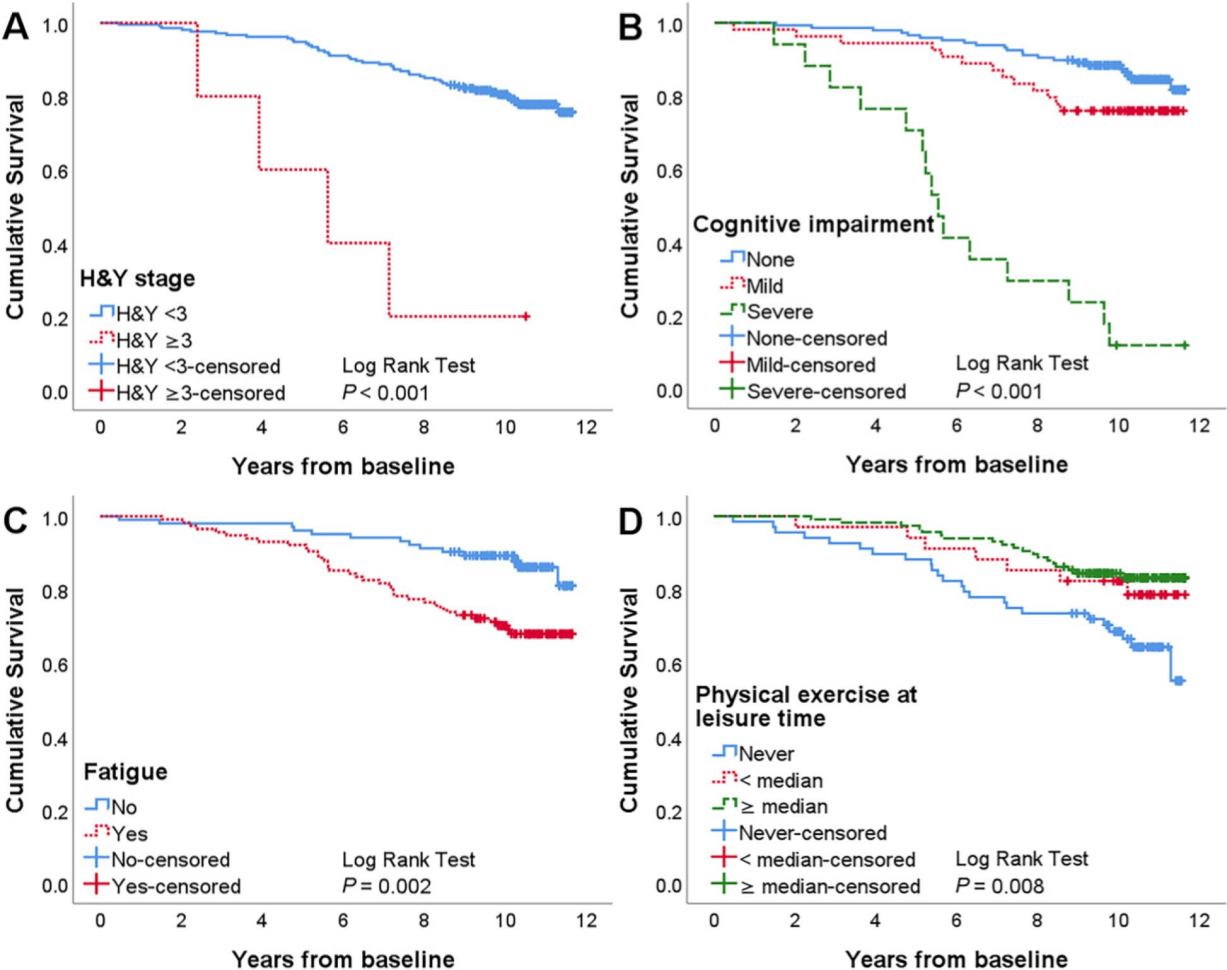
Kaplan–Meier plots of subgroups; (**a–d**) Kaplan–Meier plot displaying the effect of H&Y stage, cognitive impairment, fatigue and physical exercise at leisure time on survival in PD patients

Multivariate Cox regression confirmed that older age at onset, H&Y stage ≥ 3, and severe cognitive impairment at baseline were independent predictors of shorter survival (Table 2). We calculated the overall survival curve using the Kaplan–Meier method (Fig. 2). Kaplan–Meier plots were also used to explain the impact of H&Y stage, cognitive level at baseline, fatigue and physical exercise at leisure time on survival in PD patients (Fig. 3a–d).

During the ten-year study period, a total of 50 (22.90%) patients died (24 males, 26 females). The most common cause of death was 29 (58.00%) deaths due to respiratory diseases, of which 24 (48.00%) were pneumonia. Seven (14.00%) deaths were due to heart disease. Other causes included four (8.00%) cases of stroke, five (10.00%) cases of digestive system disease, one (2.00%) case of urinary system disease, two (4.00%) cases of suicide, one (2.00%) case of heat stroke, and one (2.00%) unknown cause (Table 3). The overall SMR was 1.32 (95% CI 0.98–1.74). The SMR was calculated as 1.13 (95% CI 0.60–1.93) for patients who opted for DBS surgery during the follow-up.

**Table 3:** Primary causes of death and their ICD-10 codes

	ICD-10 codes	Observed, No. (%)
All cause		50 (100.00)
Respiratory	J00-J99	29 (58.00)
Cardiac	I09-I25	7 (14.00)
Stroke	I67	4 (8.00)
Digestive	K00-K93	5 (10.00)
Urinary	N00-N99	1 (2.00)
Suicide	X60-X84	2 (4.00)
Thermoplegia	W85-W99	1 (2.00)
Unknown reason	R99	1 (2.00)

*ICD-10* Tenth revision of the International Classification of Diseases

## Discussion

Our work is the first to report a survival survey of PD patients in the northern Chinese mainland. We demonstrated that older age at onset, a baseline H&Y stage of 3 or higher and severe cognitive impairment were independent predictors of poor survival. It is recognized that older age of onset is a demographic indicator closely related to shortened survival. It is not difficult to understand that the incidence and prevalence of PD increase with age, while advanced age means increased clinical comorbidities and shortened remaining lifespan.

Findings regarding whether the H&Y stage independently predicts a decline in survival in PD have not been entirely consistent [6, 7]. In our study, higher baseline H&Y staging was independently associated with shorter survival. First, the H&Y scale reflects different stages of disease progression. A higher staging means further loss of dopaminergic neurons in the nigrostriatal pathway, for which itself, or its resulting dysfunction, may be related to a higher mortality rate in patients [7]. Second, an H&Y stage of 3 or higher implies involvement of posture balance. It is generally believed that posture balance disorders are less responsive to dopaminergic drugs and more related to nondopaminergic systems, which are associated with poor prognosis [4]. Unfortunately, the lack of scale data in Off medication limited our more comprehensive and accurate analysis of the disease severity to some extent.

Severe cognitive impairment at baseline was another important predictor of poor survival in this cohort (HR 6.18, 95% CI 2.75–13.88). Previous studies using the MMSE to evaluate cognitive impairment in PD patients have shown similar findings [4, 7]. Memory loss, visuospatial dysfunction and especially impaired executive function are prominent aspects of cognitive impairment in PD patients. Interestingly, in the CamPaIGN cohort study, two types of cognitive impairment were found in PD patients, and the prognoses were different between the two [26]. Executive dysfunction based on an impaired frontal lobe-subcortical loop in the early stage of the disease is regulated by the catechol-O-methyltransferase genotype and dopaminergic drugs, is not related to overall cognitive decline and dementia in the next five years, and often has a good prognosis [26]. In contrast, early defects in cognitive tasks based on posterior cortical areas, such as the temporal and parietal lobes, developed into subsequent dementia, which was related to the deposition of cortical Lewy bodies and defects in cholinergic transmitters. Such cognitive impairment had a poor response to dopaminergic drugs and predicted a decline in survival [26]. This suggests that, to guide targeted interventions in the future, we should explore whether damage in different cognitive domains differentially impacts the survival of PD patients.

We report for the first time in a univariate analysis that fatigue may contribute to an increased risk of death in PD patients. Fatigue is an independent nonmotor symptom that runs through the whole course of PD, and its incidence rate increases gradually with PD progression [27]. The meta-analysis revealed a fatigue incidence of 50% in PD patients [24], with similar findings in our study (52.80%). The FSS scale used in this study was the only scale rated as "recommended" by the Movement Disorder Society Working Group and was reliable and effective for assessing fatigue in PD patients. Previous studies have shown that PD patients with fatigue are characterized by higher age, longer course of disease and more severe motor symptoms and have a higher risk of cognitive impairment, daytime sleepiness, anxiety and depression [24]. However, Kostić et al. [28] pointed out that the occurrence of fatigue had its own internal pathological mechanisms, mainly including abnormalities of the basal ganglia-cortex pathway, especially the frontal lobe circuit, imbalance of neurotransmitters such as dopamine and 5-hydroxytryptamine, change of the hypothalamic–pituitary–adrenal axis, innervation of cardiac sympathetic denervation, and neuroinflammation, etc. Therefore, fatigue was not secondary to emotional disorders, sleep changes, or drug therapy. In this study, we did not find a significant correlation between fatigue and other included variables, except for sleep quality grade, which was excluded from the multivariate model due to its multicollinearity. It may be a risk factor for decreased survival in PD patients due to pathological changes in fatigue itself, but our sample size is insufficient to detect that it is an independent predictor in multivariate analysis, probably due to its weak effect.

This study found a correlation between a certain amount of physical exercise and better survival in univariate analysis. In terms of molecular biology, exercise intervention exerts neuroprotective effects by resisting oxidative stress and neuroinflammation and upregulating the expression of brain-derived neurotrophic factor (BDNF) and glial cell line-derived neurotrophic factor (GDNF) in the nigrostriatal pathway [29–31]. In terms of clinical benefits, exercise intervention delays motor progression and cognitive decline in PD patients and improves their physical function and sleep quality [15, 32, 33]. It is speculated that the neuroprotective effect of exercise and its clinical benefits may be the reason for the final prolongation of survival. Our study quantified physical activity by calculating MET-h/wk and suggested that exercise above the median appeared to improve survival. Similarly, a recent study found that higher intensity aerobic training appeared to improve motor symptoms more significantly than moderate intensity [34]. This suggests that, in future studies, the appropriate exercise dose should be tailored for PD patients to obtain the best symptom improvement and prognostic benefit.

This survival survey in the northern Chinese mainland gave an SMR of 1.32 with a 95% CI of over 1.0, which means that the 10-year mortality rate for PD patients is similar to that expected for the general population nationwide. Our results are similar to those in Shanghai (SMR = 0.87, 95% CI 0.59–1.25) [6] and Hong Kong (SMR = 1.10, 95% CI 0.80–1.50) [4] in southern China. There seems to be no survival difference between the south and the north. The following may have contributed to the improved survival in our cohort: (1) Atypical Parkinson’s disease, which is thought to be associated with shorter survival [35], was excluded at enrollment and during follow-up. (2) Ninety (41.30%) patients in this study who opted for DBS surgery during the 10-year follow-up period may have contributed more positive effects on survival. Previous studies have shown that DBS therapy may improve the survival of PD patients [13, 36]. (3) Our patients received regular medical follow-up from movement disorder specialists, during which potential disease complications and comorbidities might have been screened and treated, which could be beneficial to overall health. (4) Only five patients in this cohort had an initial H&Y stage ≥ 3, which might have attenuated the negative impact of advanced PD on overall survival.

Several advantages of our study are worth mentioning. To the best of our knowledge, this is the first survey on the long-term survival of PD patients in the northern Chinese mainland, which provides a basis for further understanding the disease burden of PD. Second, lifestyle factors were well documented and fully quantified for all subjects. We found that physical exercise above the median seemed to be associated with improved survival, which provided a certain basis for determining the exercise dose of PD patients. In addition, we evaluated for the first time the effect of fatigue, as determined by the FSS scale, on the survival of PD patients and identified it as a possible risk factor. A major source of the limitations of this study was a degree of selection bias. First, the subjects were from a specialist clinic rather that the community. Second, due to the absence of accurate information on death certificates from other provinces, patients living outside Dalian were excluded from the study. Third, compared with other movement disorder clinics in Dalian, more PD patients chose DBS surgery in our center. In addition, the patients in this study had a relatively young onset age. The above selection bias may lead to our underestimation of SMR and should be further explored in the future in an initial cohort of PD patients from the community. Moreover, some of the variables recorded at baseline may have changed during follow-up. Unfortunately, not enough patients have had such thorough clinical evaluations at other time points after baseline, limiting our analysis of the potential survival effects of these changes. Furthermore, our sample size may not have been sufficient to detect independent survival factors with weak effects. Finally, there was a risk of overfitting in our Cox model due to a relatively low number of events per variable studied.

## Conclusions

In this PD cohort from northern China, no significant differences were observed in the ten-year survival of patients from the general population nationwide. We concluded that older age at onset, higher baseline H&Y staging, and severe cognitive impairment independently predicted a higher risk of death. Fatigue was another indicator that may lead to a deterioration in survival. To date, none of the treatments for PD have been confirmed to delay disease progression. Identifying and understanding factors related to survival will provide a novel direction for disease modification therapy, which is expected to increase the life expectancy of PD patients.

## Supplementary Information

**Additional file 1: Table S1.** Factors without statistical significance in the univariate Cox regression model.

## Data Availability

The datasets used and/or analysed during the current study are available from the corresponding author on reasonable request. De-identifed participant data are not available for legal and ethical reasons.

## Abbreviations

BDNF: Brain-derived neurotrophic factor
CI: Confidence interval
DBS: Deep brain stimulation
FSS: Fatigue Severity Scale
GDNF: Glial cell line-derived neurotrophic factor
HAMD: Hamilton Depression Scale
HR: Hazard ratio
H&Y: Hoehn and Yahr
ICD-10: Tenth revision of the International Classification of Diseases
LEDD: Levodopa equivalent daily dosage
MET-h/wk: Metabolic equivalent hours per week
MMSE: Mini-mental State Examination
NMSQ-30: 30-Item Nonmotor Symptoms Questionnaire
PD: Parkinson’s disease
PH: Proportional hazards
PIGD: Posture instability gait difficulty-dominant
PSQI: Pittsburgh Sleep Quality Index
SD: Standard deviation
SMR: Standardized mortality ratio
TD: Tremor-dominant
UPDRS: Unified Parkinson Disease Rating Scale
