# Supplementary material for: Survival in patients with Parkinson’s disease: a ten-year follow-up study in northern China
Source: BMC Neurol. 2022 Sep 22;22:367. doi: 10.1186/s12883-022-02899-5 (PMC9494910; doi:10.1186/s12883-022-02899-5)
Supplement: Supplementary file 1 — Additional file 1: Table S1. Factors without statistical significance in the univariate Cox regression model. [file 12883_2022_2899_MOESM1_ESM.docx]

**Table S1** Factors without statistical significance in the univariate Cox regression model

| Baseline variable | Univariate Cox regression | |
| --- | --- | --- |
|  | Hazard ratio (95% CI) | *P* Value |
| Sex  Female  Male | 1.00 (reference)  1.07 (0.61–1.86) | 0.811 |
| Clinical comorbidity  No  Yes | 1.00 (reference)  1.74 (1.00–3.03) | 0.052 |
| Tremor as initial symptom |  |  |
| No  Yes | 1.00 (reference)  1.04 (0.59–1.82) | 0.904 |
| Education  Illiteracy  Primary school  Middle or high school  University or higher | 1.00 (reference)  1.25 (0.16–9.79)  1.03 (0.14–7.51)  1.23 (0.15–10.25) | 0.831  0.980  0.848 |
| Hallucinations  No  Yes | 1.00 (reference)  1.64 (0.59–4.57) | 0.341 |
| Depression  None  Mild  Moderate | 1.00 (reference)  1.22 (0.65–2.27)  2.31 (1.03–5.18) | 0.535  0.043 |
| Constipation  No  Yes | 1.00 (reference)  1.65 (0.91–2.98) | 0.100 |
| Caffeinated coffee  Never  < median (cups/day)^a^  ≥ median (cups/day)^a^ | 1.00 (reference)  0.36 (0.05–2.62)  0.33 (0.05–2.36) | 0.314  0.267 |
| Caffeinated tea  Never  < median (cups/day)^a^  ≥ median (cups/day)^a^ | 1.00 (reference)  0.70 (0.28–1.78)  0.27 (0.07–1.12) | 0.458  0.072 |
| Alcohol  Never  < median (cups/day)^a^  ≥ median (cups/day)^a^ | 1.00 (reference)  2.33 (1.04–5.23)  1.19 (0.47–3.04) | 0.040  0.712 |
| Smoking history  Never  Former  Current, at baseline | 1.00 (reference)  1.89 (0.94–3.81)  0.88 (0.27–2.87) | 0.075  0.837 |

**Table S1** Factors without statistical significance in univariate Cox regression model *(Continued)*

| Baseline variable | Univariate Cox regression | |
| --- | --- | --- |
|  | Hazard ratio (95% CI) | *P* Value |
| Smoking history, 1-pack-year increase | 1.01 (0.99–1.03) | 0.210 |
| LEDD, 100-mg increase | 1.07 (0.94–1.22) | 0.310 |
| Disease duration, y | 0.98 (0.90–1.07) | 0.674 |

^a^One cup = 150 ml.

*CI* confidence interval, *LEDD* levodopa equivalent daily dosage
